# Nuclear Factor Erythroid 2-Related Factor 2 Intervenes the Release of Neutrophil Extracellular Traps during Lipopolysaccharide-Induced Acute Lung Injury in Mice

**DOI:** 10.1155/2024/8847492

**Published:** 2024-08-29

**Authors:** Junying Lu, Guilan Xiong, Hongxiang Li, Dong Zhang, Xiaohao Zhang

**Affiliations:** ^1^ Department of Critical Care Medicine The First Hospital of Jilin University, Changchun, Jilin 130021, China; ^2^ Department of Critical Care Medicine Huazhong University of Science and Technology Union Shenzhen Hospital, Shenzhen, Guangdong 518000, China; ^3^ Department of Cardiology The Second Hospital of Jilin University, Changchun, Jilin 130021, China

## Abstract

The pathogenesis of acute lung injury is complex. Studies have demonstrated the role of neutrophil extracellular traps (NETs) in the process of lipopolysaccharide (LPS)-induced acute lung injury (ALI). However, the underlying mechanism remains unclear. In this study, the regulation of Nrf2 in the formation of NETs, which was pathogenic in LPS-induced ALI, was identified by analyzing the levels of Cit-H3, lung function, lung tissue pathology, lung wet/dry ratio, the inflammatory cells, cytokines and proteins in the bronchoalveolar lavage fluid (BALF) and in addition, the activity of lung myeloperoxidase (MPO) was also measured. Results showed that the levels of Cit-H3 measured by western blot in Nrf2-knockout (KO) mice were higher compared with the WT mice after LPS stimulation. To further investigate the NETs formation was pathogenic during LPS-induced ALI, the Nrf2-KO mice were treated with DNase I. Results showed that DNase I improved lung function and lung tissue pathology and significantly reduced lung wet/dry ratio and proteins in the BALF. Besides, DNase I also attenuated the infiltration of inflammatory cells and the cytokines (TNF-*α*, IL-1*β*) production in the BALF and the activity of lung MPO. Therefore, these results together indicate that Nrf2 may intervene in the release of NETs during LPS-induced ALI in mice.

## 1. Introduction

Acute lung injury (ALI) and its more severe form of acute respiratory distress syndrome (ARDS) is a life-threatening condition characterized by hypoxemia following diffuse alveolar damage and the disruption of endothelial–epithelial barrier [1]. Despite significant advances in the management of support, ALI/ARDS remains a high morbidity and mortality worldwide [2]. ALI/ARDS has brought a heavy burden to the global public health, but there is still no effective therapeutic strategy to diminish the severity of lung injury and the mortality among ALI/ARDS patients [3].

Neutrophils, the first line of the innate immune response, are thought to be key players in the pathogenesis of ALI/ARDS [4, 5]. Neutrophils are activated and accumulated in the lung microvasculature, interstitium, and alveolar space during infection-related ALI/ARDS and release numerous antimicrobial factors such as reactive oxygen species (ROS), proteinases via phagocytosis and degranulation, together with a novel antibacterial strategy called neutrophil extracellular traps (NETs) to kill pathogen. However, these factors are also toxic and significantly contribute to the progression of ALI/ARDS [5, 6].

NETs are unique web-like structures of histone-modified DNA strands and neutrophil-derived antimicrobial peptides, including elastase and myeloperoxidase (MPO) [6]. Citrullinated histones catalyzed by peptidylarginine deiminase 4 (PAD4) are identified as components of NETs and are produced as part of the neutrophil response to infections. Citrullinated-histone H3 (Cit-H3) co-localizes with extracellular fibrillary and web-like structures of NETs [7, 8]. NETs are released by activated neutrophils into the extracellular milieu to kill microbial agents; however, studies have found that excessive NETs are formed and displayed deleterious effects on lung injury during ALI/ARDS [9, 10]. In the study of blood transfusion-related lung injury (TRALI), a large number of NETs structures were detected in the alveoli of TRALI mice and patients, and DNase I treatment successfully inhibited the development of TRALI in mice [11, 12]. Therefore, strategies targeting excessive NETs may be beneficial in ARDS.

Studies have shown that in the process of LPS-induced ALI in mice, LPS induces excessive NETs, but the underlying mechanism is still unclear [13]. Convincing work shows that the release of NETs is dependent on ROS [14, 15], and there is now growing evidence to suggest that the release of NETs is oxidant-independent. Stimulus induces NETs rapidly before any intracellular oxidative stress could be detected [16, 17]. However, the downstream signals of the oxidant and upstream of the NETs are poorly understood [18].

Nuclear factor erythroid 2-related factor 2 (Nrf2) is a transcription factor that induces the expression of antioxidant and defense genes by interacting with antioxidant response elements (AREs). Nrf2 neutralizes ROS and is closely associated with the expression of anti-inflammatory, anti-apoptotic, and autophagy-related genes, playing an important protective effect in cells and tissues after damage [19, 20]. Numerous studies have confirmed the vital role of Nrf2 in the development of ALI/ARDS [21]. Nrf2 mainly functions through the target gene heme oxygenase-1 (HO-1) and reduces oxidative stress and inflammation during ALI/ARDS [22]. In addition, studies have shown that the Nrf2/HO-1 pathway plays a protective role in a variety of inflammatory diseases by inhibiting the expression of high-mobility group box 1 (HMBG1) [23], while recent studies have shown that HMGB1 induces the formation of NETs through Toll-like receptor (TLR) 4 and TLR9-dependent mechanisms in acute inflammation [23, 24]. ROS production and HMGB1 are all closely related to the formation of NETs. Moreover, a recent study has shown that Nrf2 regulates the activation and migration of neutrophils, playing a protective role in several functions of neutrophils [25]. Therefore, we hypothesized that Nrf2 may regulate the formation of NETs.

In the present study, we aimed to explore whether Nrf2 regulates the formation of NETs. We used Nrf2 knockout (KO) mice, demonstrating that the absence of Nrf2 in genetic KO mice strongly aggravated LPS-induced lung injury and increased the release of NETs. Then, we demonstrated that the NETs formation was pathogenic during LPS-induced ALI in Nrf2-KO Mice by treating mice with DNase I, which effectively hydrolyzed the backbone structure of NETs. Our study provides a novel perspective on the regulation of NETs formation during LPS-induced ALI.

## 2. Materials and Methods

### 2.1. Major Reagents

LPS was provided by Sigma–Aldrich (St. Louis, MO, USA). Deoxyribonuclease I (DNase I) was purchased from Roche Diagnostics (Mannheim, Germany). ELISA kits of mouse TNF-*α* and IL-1*β* were purchased from ImmunoWay Biotechnology Company (Plano, USA). Bicinchoninic acid (BCA) protein assay reagent was purchased from Beyotime Biotechnology (Nanjing, China). Minute TM total protein extraction kit for animal cultured cells and tissues was provided by Invent Biotechnologies (Eden Prairie, USA). Antibodies against Cit-H3 and *β*-actin were purchased from Cell Signaling Technology (Boston, MA, USA). A commercial assay kit for MPO was purchased from Nanjing Jiancheng Biological Reagent Company (Nanjing, China).

### 2.2. Animals and Grouping

Healthy C57BL/6 mice (aged 6−8 weeks, 18–22 g) were purchased from the Animal Centre of Basic Medical Sciences College of Jilin University (Changchun, China), Nrf2 KO mice were purchased from and Beijing Viewsolid Biotech Company. All mice were fed under the same conditions (12 hr light/dark cycle with room temperature 20−26°C). The experimental protocols were approved by the Ethical Committee Animal Centre of Jilin University.

The ALI model was established by instilling LPS (2 mg/kg, dissolved in 50 *μ*l of phosphate buffered saline) into the mice trachea after the administration of anesthesia. DNase I (2,000 U dissolved in 50 *μ*l PBS) was administered intravenously at 0 and 10 hr postinjection of LPS to degrade NETs in some mice, while the mice in the vehicle control group received 50 *μ*l PBS. About 24 hr after trachea instillation, all mice were deeply anesthetized for subsequent experiments with 1% pentobarbital sodium.

### 2.3. Measurement of Animal Lung Function

Lung function was measured by spirometer (Buxco, PFT Controller, DSI, USA) with tracheal instrumentation [26]. After being deeply anesthetized, mice were fixed on panels and intubated with the tracheal cannulation inserted into the separated trachea via the cut made in the upper trachea cartilaginous rings. Then, lung function was measured by a spirometer. After the baseline value for calm breath was recorded, parameters of lung function, including airway resistance (RI) and dynamic compliance, were assessed automatically via the spirometer.

### 2.4. Hematoxylin and Eosin (H&E) Staining

Lung tissues were washed with cold PBS and fixed in 4% paraformaldehyde, and then were embedded in paraffin sections and cut into 4 *μ*m slices. After staining with H&E, pathological changes of lung tissues were observed under light microscopy.

### 2.5. Lung Wet/Dry (W/D) Ratio

About 24 hr after LPS stimulation, left lung tissue was collected and weighed immediately to obtain wet weight. Then, lung tissue was placed in 60° oven for drying for 48 hr until the weight was constant, obtaining dry weight. Eventually, the lung W/D ratio was calculated.

### 2.6. Inflammatory Cell Counts and Protein Concentration in the BALF

Bronchoalveolar lavage fluid (BALF), collected with filterable PBS, was centrifuged for 10 min at the condition of 4°C, 3,000 rpm, and then the sedimented cells were resuspended in 100 *μ*l PBS. A hemocytometer was used to count the number of total cells, while the condition of neutrophil was conducted using the diff-quik staining. In addition, the BCA method with a protein assay kit (Nanjing, China) was used to detect the total protein concentration in the BALF according to the manufacturer's instructions.

### 2.7. ELISA Assay of TNF-*α* and IL-1*β*

The levels of TNF-*α* and IL-1*β* in the BALF were measured using ELISA kits (ImmunoWay, Plano, USA) according to the manufacturer's instructions.

### 2.8. MPO Assay

About 24 hr after LPS stimulation, lung tissues were collected and precisely weighted and were then homogenized with phosphate buffer saline. MPO activity of lung homogenate was measured according to the manufacturer's instructions.

### 2.9. Western Blotting Analysis

To quantify the release of NETs in lung tissues, the levels of Cit-H3 were tested by western blot. About 24 hr after LPS stimulation, lung tissues were collected and lysed in RIPA buffer (HyClone, Logan, UT, USA), and the protein in lung tissue was extracted using Minute total protein extraction kit (Invent Biotechnologies, Eden Prairie, USA). The same amounts of proteins (25 *μ*g protein per lane) were electrophoretically transferred onto PVDF membranes, which were separated by 12% SDS–PAGE. Then, the membranes were blocked with the blocking solution (5% fat-free dry milk) and incubated with the primary antibodies overnight at 4°C. After washing the membranes with TBST buffer for three times, the membranes were subsequently incubated with secondary antibodies for an additional 2 hr at room temperature. Finally, band signals were visualized with ECL chemiluminescent detection.

### 2.10. Statistical Analysis

All data were expressed as means ± standard deviation (SD). A one-way analysis of variance was performed by GraphPad Prism 6.0 to compare differences among groups. *P*  < 0.05 was considered statistically significant.

## 3. Results

### 3.1. Nrf2 Deletion Aggravates the Release of NETs during LPS-Induced ALI in Mice

**Figure 1:**
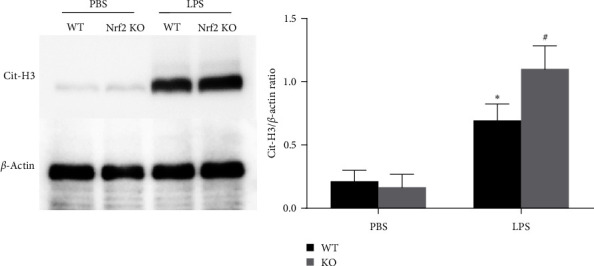
Nfr2 deletion aggravates the release of NETs during LPS-induced ALI in mice. WT and Nrf2 KO mice were stimulated with LPS or PBS, and the lung tissue was obtained 24 hr later. The levels of Cit-H3 protein in the lung tissue were determined by western blot. Values are presented as mean ± SD of three separate independent experiments.  ^*∗*^*P* < 0.05 vs. the PBS group, ^#^*P* < 0.05 vs. the WT/LPS group. *n* = 6 WT or Nrf2-KO mice/group. Nrf2 Nuclear factor erythroid 2-related factor 2; WT, wild type; KO, knockout; LPS, lipopolysaccharide; PBS, phosphate-buffered saline; Cit-H3, citrullinated histone H3.

To investigate whether Nrf2 modulates the form of NETs in LPS-induced ALI, we used Nrf2-KO mice. Given that NETs formation detected in the lung tissue lacks web-like extracellular chromatin structures, moreover, citrullinated-histone H3 (Cit-H3) co-localizes with extracellular fibrillary and web-like structures of NETs, which is another specific marker of NETs formation, we analyzed the levels of Cit-H3 by western blot. We observed that the levels of Cit-H3 were significantly higher after LPS stimulation compared to the control group in WT mice, and the levels in Nrf2-KO mice were higher after LPS stimulation compared with the LPS group in WT mice (Figure 1).

### 3.2. NETs Degradation by DNase I Attenuates LPS-Induced ALI in Nrf2-KO Mice

**Figure 2:**
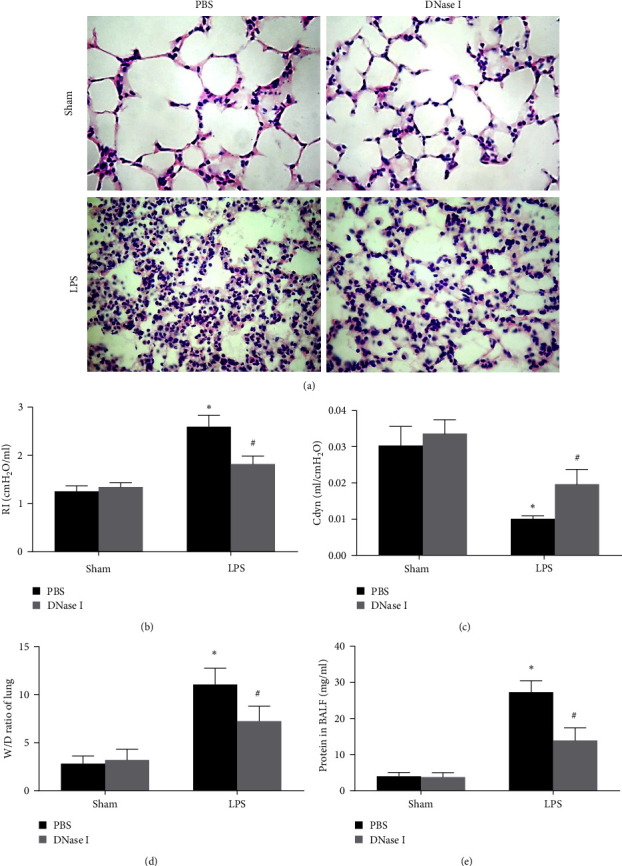
NETs regulate lung injury during LPS-induced ALI in Nrf2-KO mice. Mice were treated with DNase I 2,000 U at times 0 and 10 hr after LPS stimulation (2 mg/kg in 50 *μ*l of PBS), and lung tissues and BALF were obtained 24 hr after LPS stimulation: (a) changes in the representative histology of lung tissues (H&E staining, magnification × 400); (b) changes of lung function (RI); (c) changes of lung function (Cdyn); (d) changes of pulmonary edema (lung W/D ratio); and (e) changes of total protein concentration in BALF. Values of the experiments are presented as mean ± SD.  ^*∗*^*P* < 0.05 vs. the sham group, ^#^*P* < 0.05 vs. the LPS group. *n* = 6 Nrf2-KO mice/group.

Then, we demonstrated the NETs formation was pathogenic during LPS-induced ALI in Nrf2-KO Mice by treating mice with DNase I, which effectively hydrolyze the backbone structure of NETs. Lung tissue histopathology revealed severe destruction of the lung structures 24 hr after LPS stimulation, characterized by diffuse alveolar interstitial edema and inflammatory cell infiltration. The administration of DNase I significantly improved the lung histopathological changes (Figure 2(a)). LPS induced a significant increase in inspiratory resistance (RI), lung W/D ratio, and total proteins in the BALF and a decrease in Cdyn compared with the sham group (*P* < 0.01), the treatment of DNase I significantly improved lung function measured by RI and Cdyn and lung injury measured by lung W/D ratio and total proteins in the BALF.

### 3.3. NETs Regulated Local Inflammation of LPS-Induced ALI in Nrf2-KO Mice

**Figure 3:**
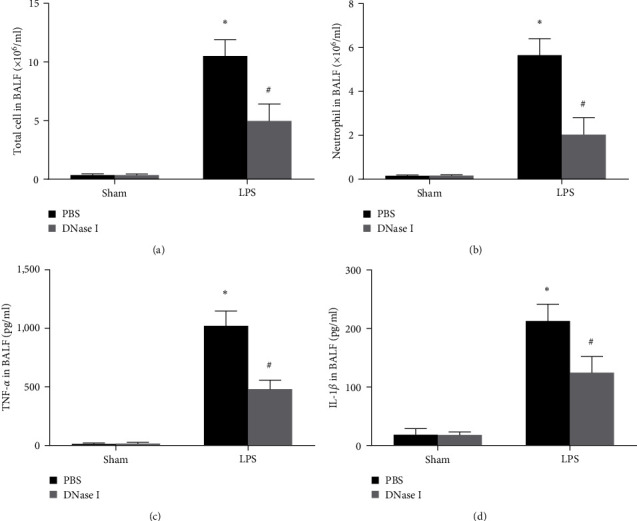
NETs regulate tissue lung inflammation during LPS-induced ALI in Nrf2-KO mice. Mice were treated with DNase I 2,000 U at times 0 and 10 hr after LPS stimulation (2 mg/kg in 50 *μ*l of PBS), and BALF was obtained 24 hr after LPS stimulation. The number of alveolar total cells and neutrophils in BALF (a and b) and cytokine production in BALF (c and d) were tested. Values of the experiments are presented as mean ± SD.  ^*∗*^*P* < 0.05 vs. the sham group, ^#^*P* < 0.05 vs. the LPS group. *n* = 6 Nrf2-KO mice/group.

To assess whether the excessive NETs formation medicated the local inflammation of LPS-induced ALI in Nrf2-KO mice, we counted the number of inflammatory cells in BALF and measured the cytokine (TNF-*α*, IL-1*β*) levels in BALF. We found that the inflammatory cell numbers and cytokine levels in BALF were very low in the sham animals, and both were markedly elevated after the stimulation of LPS and treatment with DNase I effectively reduced these levels (Figure 3).

### 3.4. NETs Regulated Lung MPO Activity of LPS-Induced ALI in Nrf2-KO Mice

**Figure 4:**
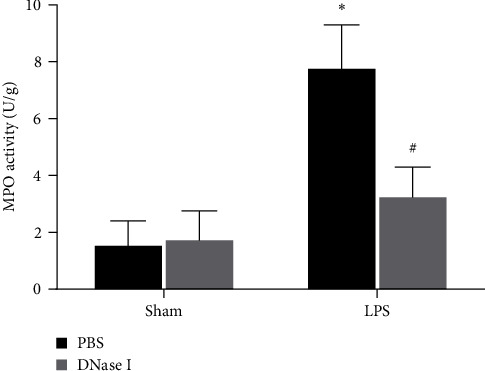
NETs regulate lung MPO activity during LPS-induced ALI in Nrf2-KO mice. Mice were treated with DNase I 2,000 U at times 0 and 10 hr after LPS stimulation (2 mg/kg in 50 *μ*l of PBS), and lung tissues were obtained 24 hr after LPS stimulation. The lung MPO activity was tested. Values of the experiments are presented as mean ± SD.  ^*∗*^*P* < 0.05 vs. the sham group, ^#^*P* < 0.05 vs. the LPS group. *n* = 6 Nrf2-KO mice/group.

Neutrophils play a key role in the development of ALI. In addition, the activity of MPO in lung tissue homogenate is an important indicator of neutrophil infiltration during ALI. To assess the effects of NETs formation on lung neutrophil infiltration during LPS-induced ALI in Nrf2-KO mice, the level of MPO was evaluated. As is shown in Figure 4, the activity of MPO was very low in the sham animals and was markedly elevated after the stimulation of LPS, and the treatment with DNase I effectively reduced the activity of MPO.

## 4. Discussion

ALI is characterized by acute diffuse and uncontrolled inflammation in the lung, which is mainly caused by a variety of direct and indirect injury factors and is associated with high morbidity and mortality [1, 2]. The pathogenesis of ALI is very complex and involves many aspects. It is currently believed that neutrophils play an important role in the pathogenesis of ARDS [4, 5]. NETs are a newly discovered bactericidal mechanism of neutrophils [6]. Recently, studies have shown that it is closely related to ALI, but the regulatory mechanism is still unclear [13, 18]. In the present study, we observed an increase in NETs formation during LPS-induced ALI in Nrf2-KO mice, demonstrated by the increased Cit-H3 levels in lung tissues revealed by western blot. Moreover, the NETs formation is pathogenic during LPS-induced ALI in Nrf2-KO mice, regulating lung injury and local inflammation, which is demonstrated by the treatment of DNase I. Our findings indicate that Nrf2 is involved in regulating the release of NETs for the first time.

NETs bind and trap various pathogens and promote the killing of them, ultimately counteracting the control of infections [4, 5, 6]. However, studies have shown that excessive NETs formation acts as danger-associated molecular pattern and are associated with inflammation and tissue injury. Studies investigating the role of NETs in ALI have shown that excessive NETs structures were detected in the ALI model induced by transfusion, LPS, and ventilator; moreover, NETs degradation by DNase I treatment had a significant impact on the degree of lung injury and inflammation induced by transfusion and LPS [11, 12, 13, 27]. In the present study, we also found that after the stimulation of LPS, NETs structures were excessively released. Our results were consistent with previous reports on the release of NETs in LPS-induced ALI mice. However, little is known about the signal regulation of NETs in LPS-induced ALI in mice.

Nrf2 functions through combining with AREs neutralize ROS, thereby playing a protective role in respiratory diseases [21]. Researchers using Nrf2 KO mice found that Nrf2 deletion was associated with high susceptibility and more severe insults in the model of ARDS, which was caused by various factors [28]. In agreement with these findings, in the current study, we demonstrated that in the mice of LPS-induced ALI, the lung damage was more severe after the deletion of Nrf2, and the levels of NETs in Nrf2-KO mice were higher than that in WT mice; moreover, we found that the degradation of NETs by DNase I significantly improved the degree of lung injury and local inflammation in Nrf2-KO mice, suggesting that the deletion of Nrf2 affects the formation of NETs. Studies show that Nrf2 by targeting AREs and HO−1, Nrf2 inhibits the production of ROS and HMGB1, playing an important protective role in ALI and other inflammatory diseases, while ROS generation is essential for the release of NETs and HMGB1 interacting with TLR4 and TLR9 and promotes the formation of NETs, and exacerbates inflammation and tissue injury [22, 23, 24]. Nrf2 may regulate the formation of NETs through ROS or HMGB1. Although we have demonstrated that Nrf2 could regulate the formation of NETs in the lung of LPS-induced ALI, we have not yet demonstrated its specific regulatory mechanism, which needs further research.

In summary, the results of this study demonstrated that the NETs released in the lung of LPS-induced ALI mice were regulated by Nrf2. A better understanding of the mechanisms of NETs formation in the mice of LPS-induced ALI is to develop the potential new treatment strategies for ALI.

## Data Availability

Data are available once receiving request.
